# Oxytocin and Psychological Factors Affecting Type 2 Diabetes Mellitus

**DOI:** 10.1155/2012/560864

**Published:** 2012-09-10

**Authors:** K. Kontoangelos, A. E. Raptis, C. C. Papageorgiou, P. C. Tsiotra, G. N. Papadimitriou, A. D. Rabavilas, G. Dimitriadis, S. A. Raptis

**Affiliations:** ^1^1st Department of Psychiatry, Eginition Hospital, Athens University Medical School, 11528 Athens, Greece; ^2^University Mental Health Research Institute, 11527 Athens, Greece; ^3^2nd Department of Internal Medicine, Research Institute and Diabetes Center, Athens University Medical School, Attikon University Hospital, 12462 Athens, Greece; ^4^Hellenic National Center for Research, Prevention and Treatment of Diabetes Mellitus and Its Complications (HNDC), Athens, Greece

## Abstract

*Background*. The aim of this study was to investigate the association of oxytocin with trait and state psychological factors in type 2 diabetic patients. *Methods*. OXT and psychological variables were analyzed from 86 controlled diabetic patients (glycosylated haemoglobin A1c (HbA1c) < 7%) from 45 uncontrolled diabetic patients (HbA1c ≥ 7). Psychological characteristics were assessed with the Eysenck Personality Questionnaire (EPQ), while state psychological characteristics were measured with the Symptom Checklist 90-R (SCL 90-R). Blood samples were taken for measuring oxytocin in both subgroups during the initial phase of the study. One year later, the uncontrolled diabetic patients were reevaluated with the use of the same psychometric instruments. *Results*. During the first evaluation of the uncontrolled diabetic patients, a statistically significant positive relationship between the levels of OXT and psychoticism in EPQ rating scale (*P* < 0.013) was observed. For controlled diabetic patients, a statistically significant negative relationship between oxytocin and somatization (*P* < 0.030), as well as obsessive-compulsive scores (*P* < 0.047) in SCL-90 rating scale, was observed. During the second assessment, the values of OXT decreased when the patients managed to control their metabolic profile. *Conclusions*. The OXT is in association with psychoticism, somatization, and obsessionality may be implicated in T2DM.

## 1. Introduction

Oxytocin (OXT) is a neurohypophysial hormone synthesized in the paraventricular and supraoptic nuclei of the hypothalamus [1]. OXT is currently attracting considerable attention, as a result of the discovery of a variety of behavioural functions it regulates, especially in the context of social interactions. A variety of behaviours, including maternal care and aggression, pair bonding, sexual behaviours, social memory, and support, as well as anxiety-related behaviour and stress coping, are modulated by brain OXT [2]. Limited data suggests a link between OXT and neuropsychiatric disorders. Of particular interest are the effects of OXT on stress induced behaviors and psychological functions. Differences in the activity of the brain OXT systems between selectively bred for high- and low-anxiety related behaviours rats may, at least partially, contribute to the opposing anxiety but not depression-relate behavior [3]. Receptors of OXT have been identified in some brain areas which have also been implicated in the pathophysiology obsessive-compulsive disorder (OCD) [4, 5]. Recently, a significant correlation was found between plasma OXT and depressive symptoms [6].

In schizophrenia, a morphometric evaluation of neurophysin immunoreactivity in the brain of untreated schizophrenic patients revealed an altered OXT function [7]. Oxytocin and its receptors exist in areas of the brain implicated in the symptoms of schizophrenia such as the nucleus accumbens and the hippocampus. Laboratory demonstrated that peripherally administered oxytocin produces reversal of prepulse inhibition deficits receptor antagonist MK-801, a finding consistent with the effects of atypical antpsychotics. Preclinical findings support an antipsychotic role of oxytocin [8]. In patients with addiction towards substances including opiates and cocaine, OXT could be involved in the development of tolerance and dependence [9], while in posttraumatic stress disorder (PTSD), administration of intranasal OXT was shown to reduce the memory retrieval and conditioned response in PTSD patients [10]. Regarding diabetes mellitus type 2 (T2DM), OXT is involved in glucose homeostasis by increasing glycogenolysis [11] as well as glycogenesis [12].

Accumulating evidence indicates a role for oxytocin in the regulation of food balance and in leptin control, as well as its stimulation of glucose uptake in rat skeletal muscles and cardiomyocytes. Therefore, strategies aimed at increasing the activity of the cardioprotective genes in diabetes may be effective in preventing cardiac dysfunction [13].

It has been found that OT can increase feeding behavior. Experiments in dogs made diabetic with streptozotocin demonstrated that INS injections enhanced OT secretion, whereas elevated plasma glucose levels fell to control values. These findings indicated that an OT link to INS (sensitive tissues such as adipocytes, skeletal and cardiac muscles, the pancreas, and endothelial cells) mediated glucose uptake in the body. Furthermore, it was recently shown that OT treatment increased glucose uptake in skeletal muscles precursor cell lines [14].

 Indicating an increase in the synthesis of hormone. Increased oxytocin production was also shown in type 1 diabetic patients in the absence of osmolality changes [15].

 OXT secretion is regulated by CD 38, a transmembrane receptor with ADP-ribosyl cyclase activity [16]. Mutations in CD 38 gene in diabetic patients could contribute to the development of T2DM via genetic defects in the cell/liver glucose transporter gene (GLUT2) [17]. Several authors have attempted to identify a line between personality type and the problems associated with the management and control of diabetes in adults and children [18].

Bradley and Cox reported a significant positive correlation between the average blood glucose and level and the extraversion score on the Maudsley personality inventory [19]. Personality could be a causal factor in a disease through a variety of very different types of mechanisms. For example, personality could lead to disease through unhealthy behaviours [20]. Personality typically refers to an integrated pattern of thinking, feeling, and behaving that varies among individuals, but it is substantially stable within an individual [21].

The importance of psychosocial issue in diabetes has been recognised since before the disease itself was well understood. For example, hormonal disregulation associated with depression may contribute to glycemic disregulation and increasing the severity of diabetes mellitus. Symptoms of some psychological disorders may contribute to the disruption of self-care routines and to a cycle of deteriorating physical and emotional well-being [22].

Eysenck's doctrine proposes a quantitative-dimensional model of human personality [23–26]. His personality schema, as a result of factor analytic studies [19], postulates three independent, biologically based dimensions of personality, psychoticism/socialization, neuroticism/stability and extraversion/introversion, and a lie scale measuring the tendency to fake good [24, 26].

Neuroticism is related to the experience of negative affect, emotional unstableness, depressive symptomatology, obsessiveness, and anxiety-based problems; extraversion to positive affect, sociability, activeness, dominant behaviour, assertiveness, expressiveness, cooperativeness, irresponsibility, and risk taking; psychoticism to antisocial behaviour, impulsiveness, aggressiveness, solidarity, lack of empathy/emotional insensitivity, and nonconformity, but also to creativity; the lie scale to social desirability and socialization [26].

The exact nature of the underlying mechanisms remains unclear. There is very limited data regarding the connection between OXT, T2DM, and neuropsychiatric disorders.

In view of the above considerations, it seems to be converging evidence indicating an implication of OXT and psychopathological parameters in the pathophysiology of T2DM, the nature of which remains complex and not definitive. Consequently, an investigation of the link between psychopathological configurations and OXT in controlled type 2 diabetic patients (HbA1c < 7%) as compared to uncontrolled diabetic patients (HbA1c ≥ 7) would be justified as well as needed. The aim of this study is the exploration of this relation and the influence of diabetic profile in OXT and the psychometric factors

## 2. Material and Methods

The present study was conducted at the Attikon University General Hospital, and the sample was randomly selected from the Diabetic Centre of the 2nd Department of Internal Medicine-Research Institute and Diabetes Center of Athens University Medical School. Our study included patients suffering from T2DM. Some were excluded from the study: patients with malignant diseases, endocrinological syndromes, known peripheral arteriopathy, coronary disease, kidney deficiency, patients with amputations, existent psychopathology, and those under treatment with antidepressants, tranquilizers, antipsychotic, and anticonvulsant medication. The study was approved by the science and Ethics Committee of the “Attikon” University General Hospital, and all the participants were informed about the purposes of the study and gave their written consent. The sample consisted of 131 diabetic patients, 55 females with mean age of 62, 9 ± 9, 64 years and 76 males with mean age of 64.8 ± 10.17 years.

The mean age of the subjects was 64 ± 9.95 years and the vast majority had poor educational background. Regarding the occupational status, 30.5% were pensioners, 37.4% were housewives, 7.6% were civil servants, and 24.4% were self-employed. The mean duration of diabetes for all participants was 11.9 ± 8.73 years with uncontrolled patients' mean being 12.6 ± 8.31 years and controlled 11.5 ± 8.96 years (Table 1).

The mean value of HbAic was 5.96 in controlled diabetic patients while in uncontrolled was 8,21.

The sample was divided in to two groups, the group A which consisted of 86 (65.6%) controlled diabetic patients (HbA1c < 7%) and the group B which consisted of 45 (35.4%) uncontrolled diabetic patients (HbA1c ≥ 7%). The group A, consisted of 36 female and 50 male participants, while the group B consisted of 20 female and 25 male participants, respectively. During the initial phase of the study (T0), blood samples were taken for measuring OXT serum levels. One year later (T1), the uncontrolled diabetic patients were reevaluated with the use of the same psychometric instruments and with the identical blood analysis. During the intermediate year, an intensive effort to improve their metabolic profile was performed with frequent appointments with the hospital dieticians, diabetologists, and adjustments of their medications.

From the initial sample of 45 uncontrolled diabetic patients, 4 died from natural causes, while 10 withdraw from the study for other personal and family reasons such as relocation. Finally, 31 uncontrolled diabetic patients were reassessed. The reevaluated sample consisted of 17 male and 14 female participants. Ten of these 31 uncontrolled patients had already managed to improve their metabolic profile (HbA1c < 7%) in the preceding year between phase 1 (T0) and phase 2 (T1) of the study.

 During the initial evaluation, all participants were assessed with two psychometric questionnaires: for measurement of the Eysenck's personality traits, the Greek standardization [27] of the EPQ [24] was applied. The questionnaire follows a dichotomous approach (yes/no answers), and the dimensions evaluated are extraversion (EPQ-E), neuroticism (EPQ-N), psychoticism (EPQ-P), and lie (EPQ-L).

SCL-90 is a 90-item self-rating instrument constituted by nine subscales, namely, depression, hostility, anxiety, phobic anxiety, obsessive compulsive, interpersonal sensitivity, somatisation, paranoid ideation, and psychoticism [28]. Each item of the SCL-90 is rated by the subject evaluated on a five-point scale of distress from zero (none) to four (very high levels), in reference to how much discomfort the particular problem has caused to him during the past 7 days, including the current day. SCL-90 assesses the psychological symptomatology patterns of an individual at present and not underlying personality traits [29].

OXT levels were measured in the serum of all the patients by use of a standard commercial ELISA (enzyme-linked immunosorbent assay) according to instructions of the manufacture (Phoenix Pharm, Europe GMBH, Karlsruhe, Germany). The sensitivity of the method was 0.08 ng/mL, while intra-assay and interassay coefficients of variation were 5–10% and <15%, respectively.

All samples were used undiluted and were all inside the standard curve range (0.01 ng–100 ng/mL). The assay recognizes only oxytocin peptide and has no cross-reactivity with any other peptide (e.g., vasopressin, somatostatin, CRF, etc.).

Although serum oxytocin was used to be measured using RIA assays, lately, there were studies using Elisa kits highly specific for oxytocin measurement [30, 31]. Furthermore, RIA and Elisa immunoassays are not comparable because of differences in antibody epitope recognition. All samples were measured twice.

### 2.1. Statistical Analysis

Continuous variables are presented as mean values with standard deviations, whereas frequencies and percentages are given for the categorical variables. Association between the metabolic profile and each of the continuous variables is explored through the Students' *t*-test under the assumption of normally distributed variables and equality of variances. The hypothesis tests that compare the scores of EPQ and SCL-90 scales between the first and second evaluation are based on paired *t*-test when assuming normally distributed variables. In the case of small sample size or asymmetric distributions, the corresponding nonparametric tests are performed, such as Mann-Whitney test for *t*-test and Wilcoxon test for paired *t*-test. Linear relationships between continuous variables are measured through the Pearson correlation coefficients for normally distributed variables or the Spearman correlation coefficients otherwise. Associations between categorical variables are examined using *χ* ^2^ test. Multiple logistic regression analysis is performed to examine the association between the metabolic profile, OXT and EPQ, and SCL-90 scale. The statistical test presented here is two-tailed and compared with the statistical level of 5%. All characteristics are presented as mean ± SD for continuous variables or percentages (%) for categorical variables.

## 3. Results

### 3.1. First Evaluation T0

**Figure 1:**
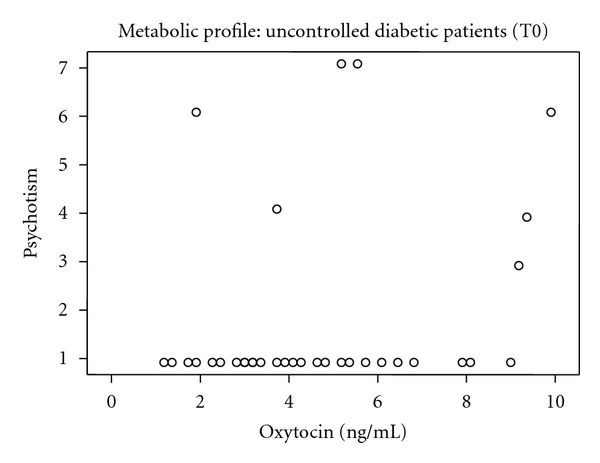
Psychotism and oxytocin in the uncontrolled diabetic patients.

In the uncontrolled diabetic patients, there is a statistical significant positive relation between OXT and psychotism in the EPQ (*r*_sp_ = 0.374, *P* = 0.013), which means that if the values of OXT increase this will increase also the values of psychotism (Figure 1).

**Figure 2:**
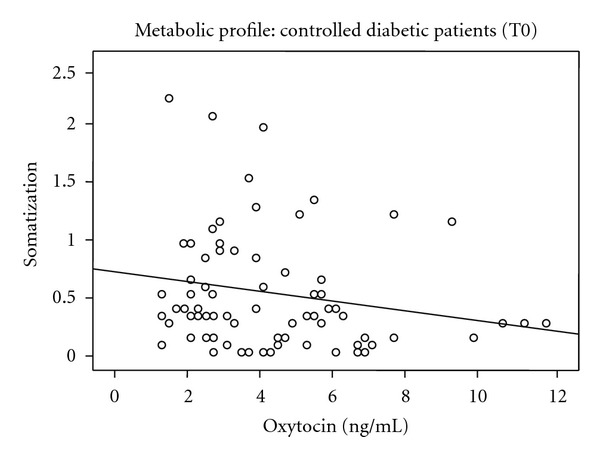
Oxytocin and somatization of SCL-90 scale in controlled diabetic Patients.

**Figure 3:**
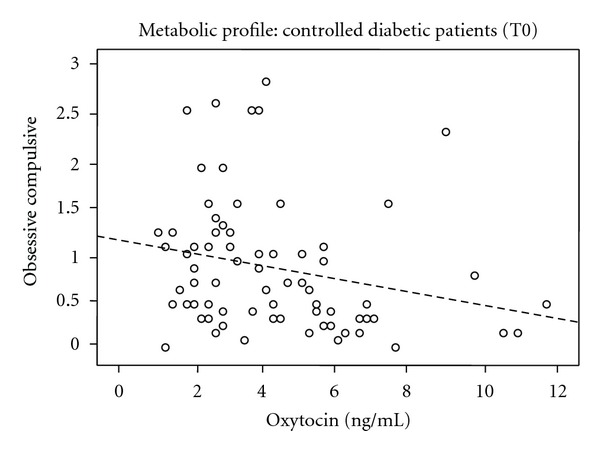
Oxytocin and obsessive compulsive of SCL-90 in controlled diabetic patients.

Regarding the correlation between the SCL-90 and OXT, there is a statistical significant negative correlation in the controlled diabetic patients between OXT and the subscales of somatization (*r*_sp_ = −0.245, *P* = 0.030) (Figure 2) and obsessive compulsive (*r*_*p*_ = −0.228, *P* = 0.047) (Figure 3).

### 3.2. T1 Second Evaluation

During the second evaluation, the values of OXT decreased when the uncontrolled patients managed to control their metabolic profile. Although this failed to reach significance levels, a clear tendency towards this direction was observed (*P* = 0.575).

Regarding the psychometric tools, the scores of psychotism, neuroticism of EPQ reduced, but these differences in scores fail to reach significance levels. In the SCL-90 scale, all the scores in the subscales reduced, but only the subscales of interpersonal sensitivity and psychotism reduced in significance levels (*P* = 0.028, *P* = 0.024).

## 4. Discussion

 In our study, during the first evaluation of the uncontrolled diabetic patients, a statistically significant positive relationship between the levels of OXT and psychotism in EPQ rating scale was observed. For controlled diabetic patients, a statistically significant negative relationship between OXT and somatization as well as obsessive-compulsive scores in SCL-90 rating scale was observed. During the second assessment, the values of OXT decreased when the patients managed to control their metabolic profile although this result failed to reach the significance level.

According to Eysenck [23], psychotism represents individuals who are solitary, emotionally cold, impersonal and hostile, and with poor social relations. On the other hand, OXT seems to play an important role in initiating and maintaining complex social behaviors [32]. Hence, the observed positive association between OXT and psychoticism appears to be paradoxical. However, a range of psychophysiological studies indicate that moderately high psychotism is associated with creativity, whether creativity is measured by achievements or laboratory tests, or it is measured in psychosis-prone individuals [29, 33, 34].

Consistent with the conception that psychoticism is associated with creativity/achievement seem to be the findings of Brunas-Wagstaff et al. [35]. They investigated the relationship between functional impulsivity, as conceptualized by Dickman [36] and the personality traits of psychoticism, extraversion, and neuroticism and found a positive correlation between functional impulsivity and psychoticism. Considering that psychoticism is generally regarded as a negative trait, whilst functional impulsivity is described in positive terms, these investigators concluded that individuals scoring high on psychoticism tend to engage in personally beneficial behaviours, even if detrimental to others or to society. In this sense, Charlton [37] proposed that the elite scientific education and training institutions ought to select their students based not only on the IQ levels, but also on the levels of creativity by taking into account the degree of psychoticism. In this sense, it is reasonable to assume as legitimate the obtained positive association between oxytocin and psychoticism. We found in our study that OXT correlates negatively with somatization and obsessive-compulsive scale in SCL-90 in regulated diabetic patients. In a previous study, Taylor et al. found no correlation between OXT and the scale of SCL-90 when they studied 73 postmenopausal women. However, they observed a correlation between OXT and women who experienced severe stress in social transactions [38].

Furthermore, in a study of 7 individuals who were diagnosed as OCD according to DSM-IV [39], no patient showed any change in their OCD symptoms despite intranasal OXT administration [40]. These results are in conflict with Ansseau et al. (1987) who reported improvement in symptoms of a patient who was treated with intranasal OXT [41]. Evidence also supports the theory that OXT potentially plays an important role in the pathogenesis of certain forms of OCD. This is nicely exemplified by experimental models in which administration of OXT can increase head engagement in mice [42]. Perhaps such behaviors may resemble repetitive behaviors of hand washing and cleanliness observed in people with OCD [43].

Intranasal OXT was found to suppress anxiety responses and to mediate the positive and buffering effects of social support [44]. Central oxytocin has been shown to promote numerous social behaviours to attenuate hormonal stress resposiveness of the HPA axis and to decrease anxiety [45].

Given the potential ability of OXT to reduce the levels of anxiety, change cognitive functions, and promote positive social relationships, investigators considered that some symptoms of depression (social withdrawal, appetite disturbance, low sexual interest, and stress response) may be associated with OXT. In addition, a small number of studies suggest low levels of OXT in depressed patients. Recent data have reported a significant negative correlation between plasma OXT levels and symptoms of depression and anxiety, in 25 patients with major depressive disorder [46]. However, abnormal values of OXT levels in depression require further investigation.

Magnocellular hypothalamic neurons are capable of synthesizing proteins within their dendrites, while the axonal compartment appears to lack synthesizing proteins capacity, despite the presence of mRNAs encoding or OXT. It is thus conceivable that separate and possibly independent neuropeptide synthesis in the sonata (for peripheral secretion and somatic release) and in dendrites (for dendritic release) contributes to independent release patterns which make measurements of neuropeptides in plasma an unreliable guide to changes within the brain.

There is no doubt that the role of the blood brain barrier varies depending on physiological versus pharmacological conditions. The former provide barrier, the endogenous neuropeptides cannot penetrate in physiologically relevant quantities, and the more so as neuropeptide concentrations in the extracellular fluid of the brain may be orders of magnitude higher than in plasma thus excluding the need of a blood to brain transport. Compared to peripheral secretion into systemic circulation, little is known about central release patterns of neuropeptides such as OXT including stimuli, mechanisms, and consequences. Generally findings based on measurements in plasma and CSF are not a reliable guide to neuropeptide changes in the extracellular fluid of distinct brain areas [47].

Moreover, OXT might be related with impairment of HPA (hypothalamus-pituitary-adrenal) axis in conjunction with the neurotransmitters that act at this level [48].

 Several studies have focused on the effect of type 2 diabetes mellitus on hypothalamic-pituitary-adrenocortical (HPA) axis functioning [49]. A study using salivary cortisol measures observed elevated evening levels in TD2M [50]. Cortisol levels among individuals with diabetes were shown to be associated with glycemic control, further suggesting that HPA axis dysregulation is linked to T2DM [51]. Neuroendocrine studies demonstrate increased responsiveness of the HPA axis activity in TD2M. For example, a stronger elevation of cortisol occurs after CRH administration [52].

Evidence for reduced feedback inhibition in the dexamethasone (DEX) suppression test has been found in some instances but not all. HPA hyperactivity and declarative memory deficits are present in TD2M. Both alterations may reflect the negative impact of poor glycemic control on the hippocampal formation [53].

 We have additionally found in our study that during the second evaluation, levels of OXT decreased in those individuals who managed to reduce their HbA1c levels, but this reduction although tends to was statistically significant.

 Oxytocin is potentially involved in glucose homoeostasis by increasing glycogenolysis as well as gluconeogenesis [54]. In addition, since OXT has been implicated in the pathophysiology of different neuropsychiatric disorders [55], the research of the implication between diabetes mellitus and psychological parameters might contribute to the progress of psychopathology research and lead to new and more accepted treatment interventions.

## 5. Limitations

Finally, there are certain limitations that should be acknowledged. The cross-sectional nature of this study precludes causal inference but provides valuable directions for future investigations. Due to the high dropout rate the sample size was diminished and that affected greatly the robustness of our statistical analysis.

## 6. Conclusions

In conclusions, taken together the present results provide evidence that OXT is closely related to the course and treatment of type 2 diabetes. However, the specific implications of the research on clinical practice remain uncertain, but it is likely that additional work in this area will further demystify and validate essential mechanisms involved in the pathogenesis of type 2 diabetes. Such an approach offers also the promise to help treatment selection by providing enhanced vocabulary for discussing concepts central to diabetes treatment.

## Figures and Tables

**Table 1:** Demographic characteristics of the sample.

	Total (*n* = 131)		Controlled (*n* = 86)		Uncontrolled (*n* = 45)	
Age	64.0	(±9.95)	64.2	(±10.94)	63.6	(±7.84)
Education						
Elementary	106	(80.9%)	71	(82.6%)	35	(77.8%)
Secondary	8	(6.1%)	5	(5.8%)	3	(6.7%)
Highest	17	(13.0%)	10	(11.6%)	7	(15.6%)
Occupation						
Housekeeping	49	(37.4%)	30	(34.9%)	19	(42.2%)
Pensioner	40	(30.5%)	26	(30.2%)	14	(31.1%)
Civil servant	10	(7.6%)	8	(9.3%)	2	(4.4%)
Self employed	32	(24.4%)	22	(25.6%)	10	(22.2%)
Duration of diabetes mellitus type 2	11.9	(±8.73)	11.5	(±8.96)	12.6	(±8.31)

(Parenthesis display the standard deviations).
